# Initial economic damage from the COVID-19 pandemic in the United States is more widespread across ages and geographies than initial mortality impacts

**DOI:** 10.1073/pnas.2014279117

**Published:** 2020-10-20

**Authors:** Maria Polyakova, Geoffrey Kocks, Victoria Udalova, Amy Finkelstein

**Affiliations:** ^a^Center for Health Policy, Department of Medicine, Stanford University School of Medicine, Stanford University, Stanford, CA 94305;; ^b^Department of Economics, Massachusetts Institute of Technology, Cambridge, MA 02142;; ^c^Enhancing Health Data Program, Demographic Directorate, US Census Bureau, Washington, DC 20233

**Keywords:** COVID-19, excess all-cause mortality, economic damages

## Abstract

The economic and mortality impacts of the COVID-19 pandemic have been widely discussed, but there is limited evidence on their relationship across demographic and geographic groups. We use publicly available monthly data from January 2011 through April 2020 on all-cause death counts from the Centers for Disease Control and Prevention and employment from the Current Population Survey to estimate excess all-cause mortality and employment displacement in April 2020 in the United States. We report results nationally and separately by state and by age group. Nationally, excess all-cause mortality was 2.4 per 10,000 individuals (about 30% higher than reported COVID deaths in April) and employment displacement was 9.9 per 100 individuals. Across age groups 25 y and older, excess mortality was negatively correlated with economic damage; excess mortality was largest among the oldest (individuals 85 y and over: 39.0 per 10,000), while employment displacement was largest among the youngest (individuals 25 to 44 y: 11.6 per 100 individuals). Across states, employment displacement was positively correlated with excess mortality (correlation = 0.29). However, mortality was highly concentrated geographically, with the top two states (New York and New Jersey) each experiencing over 10 excess deaths per 10,000 and accounting for about half of national excess mortality. By contrast, employment displacement was more geographically spread, with the states with the largest point estimates (Nevada and Michigan) each experiencing over 16 percentage points employment displacement but accounting for only 7% of the national displacement. These results suggest that policy responses may differentially affect generations and geographies.

## Significance

A full picture of the COVID-19 pandemic requires information on how its impact on lives and on livelihoods relates across different groups. We therefore estimate excess all-cause mortality and employment displacement in April 2020 nationally and separately by state and by age group. Initial economic damages from the pandemic are more widespread across groups than deaths, which were primarily concentrated in a few states and among the oldest old. While the two states with the largest mortality increase account for about half of national excess mortality, the two most economically affected states account for only 7% of national economic damages. These findings suggest that policy responses to contain the pandemic involve trade-offs across different demographic and geographic groups.

The economic and mortality impacts of the COVID-19 pandemic have been widely discussed, yet most studies to date have examined these effects separately from each other (1–10), with little systematic evidence on their relationship across different groups. As the public discussion of the impact of the pandemic on lives and livelihoods evolves, it is important to understand how the groups most affected by the pandemic’s economic impacts compare to those most affected by its health impacts. Focusing on the initial impact of the pandemic in April 2020, we therefore estimate the pandemic’s economic damage and its mortality impact and compare results by state and by age group.

We measure economic damages and mortality impacts relative to what we predict would have happened in April 2020 in the absence of the pandemic. We define economic damages as decreases in the employment-to-population ratio, which measures the fraction of people in a well-defined group currently working full time or part time. We focus on all-cause mortality rather than COVID-19 deaths for our measure of the pandemic’s impact on health. All-cause mortality has two important advantages relative to COVID-specific death counts. First, it is not contaminated by measurement error in the choice of what to label a “COVID” death as opposed to a non-COVID death; this would be particularly problematic if measurement error varies across groups. Second, all-cause mortality captures both direct and indirect mortality effects from the pandemic. Indirect effects could be negative [such as increases in mortality attributable to reduced utilization of routine and emergency healthcare services (11–13)], positive [such as declines in mortality due to reduction of motor vehicle use (14, 15)], or a priori ambiguous [such as the ongoing debate over the mortality effects of an economic downturn (16–19)].

We use the same approach to compute excess all-cause mortality and to compute excess declines in the employment-to-population ratio nationally—and separately for each US state and age group—in April 2020: We compare the observed outcome to the outcome predicted based on monthly historical data from 2011 to 2019 (*Materials and Methods*). Across age groups 25 y and older, excess mortality was negatively correlated with economic damage, with the mortality impacts concentrated among the elderly—particularly the oldest old—and the economic impacts concentrated among younger workers. Across states, employment displacement was positively correlated with excess mortality, but economic damages were much more widespread. These findings suggest that policies aimed at preserving health or reducing economic damage may involve important trade-offs across groups.

## Results

### Nationally.

**Fig. 1.**
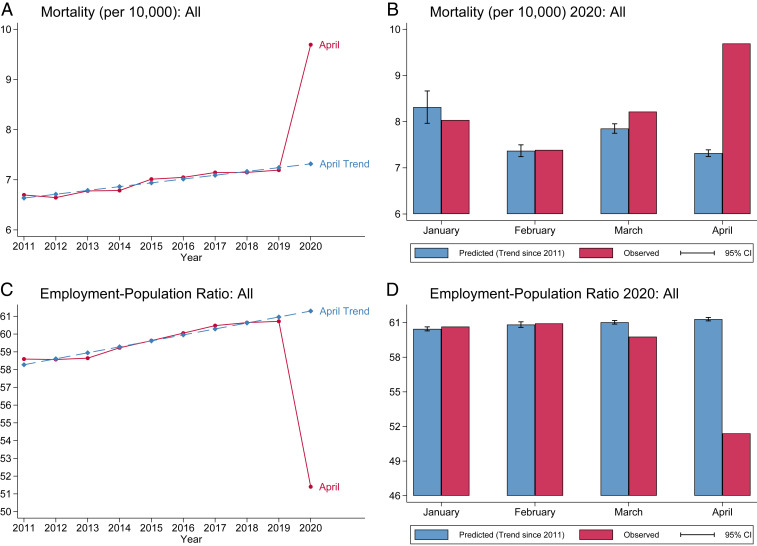
Time series of mortality and economic outcomes. These figures show time series for the monthly mortality rate per 10,000 (*A* and *B*) and employment–population ratio (*C* and *D*). *A* and *C* show the time series of the indicated variable in April of each year. *B* and *D* compare predicted outcomes to the point estimates for observed outcomes for each variable in 2020; predictions for each variable are calculated using a linear time trend with month fixed effects during the preperiod 2011 to 2019. Mortality data use CDC weighted death count. Mortality data include individuals of any age while economic data include individuals 16 y and older. Heteroskedasticity-robust SEs for calculating confidence intervals within each month are computed with clustering by year.

There was a sharp rise in national all-cause mortality in April 2020 relative to predicted mortality (Fig. 1*A*). Historical trends predict 7.3 all-cause deaths per 10,000, while we observed 9.7 per 10,000. This corresponds to 2.4 excess deaths per 10,000 (about a 33% increase in all-cause national mortality). The gap between observed and predicted mortality was not present in January or February 2020 but is already visible—at a much smaller magnitude—in March 2020 (Fig. 1*B*). Reported COVID-19 deaths make up ∼75% of the estimated all-cause excess mortality (*SI Appendix*, section 2.3). We also looked at excess mortality separately for specific causes of death, finding substantial excess mortality from heart disease and substantially lower mortality due to unnatural causes (accidents, homicides, and suicides) and to cancer (*SI Appendix*, section 7.2).

Analogously, there was a sharp decline in the employment-to-population ratio in April 2020—our measure of economic damages—relative to the prediction (Fig. 1*C*). Historical trends predict that 61.3% of 16+-y-olds would have been working in April 2020. By contrast, the observed employment-to-population ratio in April 2020 is 51.5%. This corresponds to a national excess decline in the employment-to-population ratio of 9.9 per 100 individuals. As with mortality, the effect is concentrated in April, with some gap opening in March and substantially smaller differences between predicted and observed employment-to-population ratio in January and February 2020 (Fig. 1*D*).

### By Geography.

**Fig. 2.**
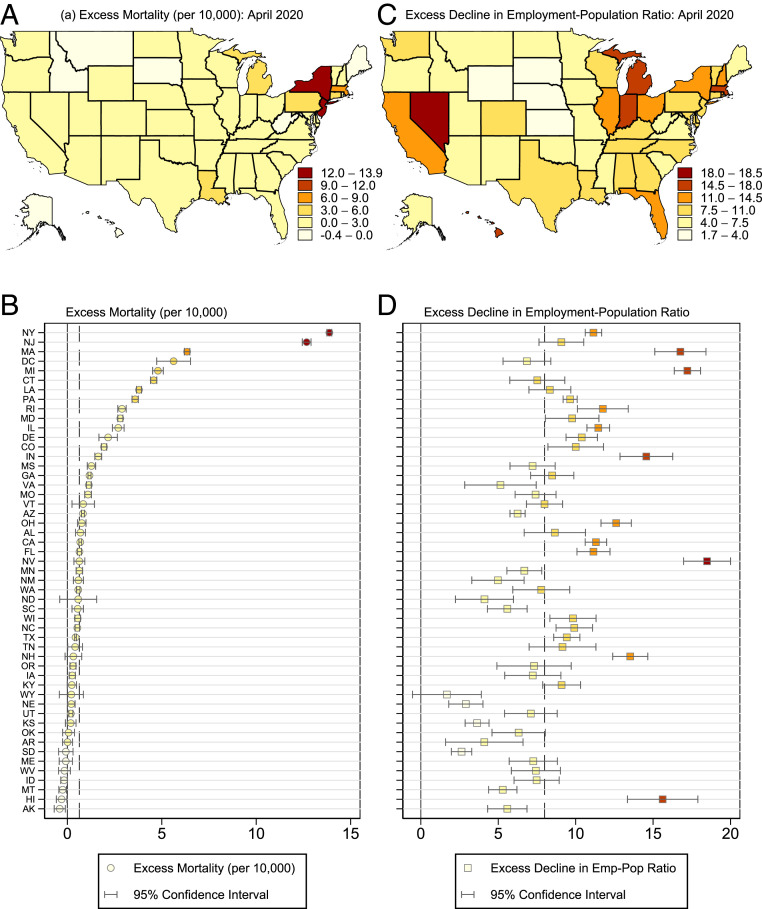
Geographic heterogeneity in outcomes by state: April 2020. These figures show deviations from expected (*A* and *C*) mortality and (*B* and *D*) employment–population ratios within each state in April 2020. For dot plots in *C* and *D*, states are ordered in descending order of their excess mortality. Dotted lines show the median of each outcome across states in April 2020. CPS data include all individuals 16 y and older and CDC data include all individuals of any age. Mortality data use CDC weighted death count estimates. Predicted values are calculated using a linear time trend with month fixed effects with preperiod 2011 to 2019. Annual population counts to calculate mortality come from the Census Bureau in each state from 2011 to 2019. Heteroskedasticity-robust SEs to construct 95% confidence intervals are computed with clustering by year.

The increase in all-cause mortality and the decline in employment-to-population ratio was markedly unequal across different geographic locations. New York and New Jersey were stark outliers in excess all-cause mortality, experiencing more than 10 excess deaths per 10,000 individuals, compared to the median excess all-cause mortality across states of 0.64 excess deaths per 10,000 (Fig. 2 *A* and *B*). In relative terms, the increase in all-cause mortality was 207% in New York and 179% in New Jersey. The health damages were highly geographically concentrated—with New York and New Jersey together accounting for 49% of the national change in all-cause mortality in April.

The situation looks remarkably different for economic damages. While there is substantial geographic heterogeneity in the economic impacts of COVID, the distribution of economic damage is far more widespread than the excess mortality impacts. The point estimates indicate that every single state experienced economic damage; this damage is statistically significant at the 5% level for all states except Wyoming. The median employment displacement across states was 7.8 percentage points. The states with the largest point estimates—Nevada and Michigan—each experienced employment displacement of over 16 percentage points, or approximately a 30% decrease in employment.* However, they accounted for only 7% of the national employment displacement (*SI Appendix*, section 5 provides more detail on these and alternative calculations).

**Fig. 3.**
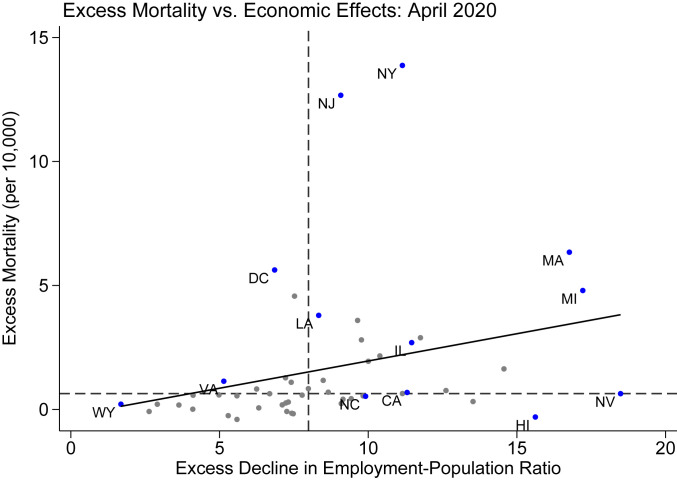
Excess mortality and economic damage comparison by state. This figure compares excess mortality and the economic impacts of COVID-19 in April 2020; each point is one state. Economic measures are calculated using the IPUMS CPS microdata and mortality measures are calculated using aggregate CDC data. Excess amounts are calculated by comparing observed values in April 2020 to predicted values. Predicted values are calculated using a linear time trend with month fixed effects; the preperiod is 2011 to 2019. The solid line shows the line of best fit from an unweighted regression. Dashed lines show the median value of excess mortality and the excess decline in employment–population ratio.

Fig. 3 plots excess mortality against economic damage by state, with the line of best fit superimposed. Early health and economic consequences of the pandemic are positively correlated across states, although the relationship is far from perfect: The line of best fit has a slope of 0.22 and is significant at the 5% level (*P* = 0.017). Some states that experienced large economic damages early on in the pandemic—such as Nevada and Hawaii—did not experience a substantial increase in mortality. Other locations—such as Washington, DC and Virginia—experienced above-median excess mortality but below-median economic damage. Industry composition appears to be an important factor in either mitigating or exacerbating the economic effects of the pandemic, as larger economic damages are observed in states with higher exposure to tourism, entertainment, and food industries (*SI Appendix*, section 6).

### By Age.

**Fig. 4.**
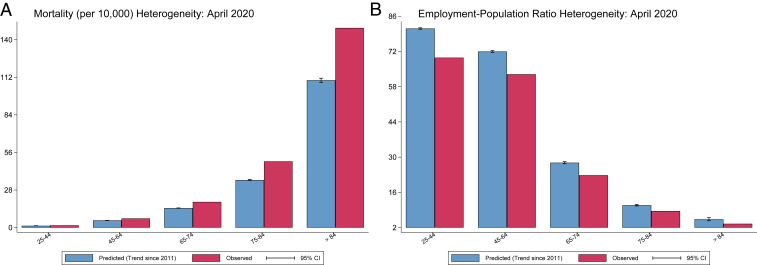
Predicted vs. observed outcomes by age group. These figures compare predicted and observed (*A*) mortality per 10,000 and (*B*) employment–population ratio, both in April 2020, for the age groups for which the CDC publishes recent weekly death counts, using point estimates for observed outcomes. The employment–population ratio is calculated using the IPUMS CPS microdata and mortality measures are calculated using aggregate CDC data by age group. Predicted values for each outcome are calculated using a linear time trend with month fixed effects; the preperiod is from 2011 to 2019. Heteroskedasticity-robust SEs to construct 95% confidence intervals are computed with clustering by year.

The increase in all-cause mortality and the decline in employment-to-population ratio affected markedly different age groups (Fig. 4). Once again, excess mortality is more concentrated while economic damages are more widespread. Excess mortality is monotonically increasing in age. Among age groups for which we observe both economic and mortality outcomes, the youngest age group (25 to 44 y) experienced excess all-cause mortality of 0.3 per 10,000 (a 22% increase), while the oldest age group (85+ y) experienced excess mortality of 39.0 per 10,000 (a 35% increase). This age group (85+ y) accounted for 3% of the population (out of those 25+ y) but 34% of the excess deaths.

The pattern is the opposite for employment displacement, with economic damages falling monotonically in age, which is not surprising given the low rates of employment at older ages. Younger adults (25 to 44 y) experienced employment displacement of 11.6 percentage points, compared to 1.8 percentage points for the oldest age group (85+ y); 25- to 44-y-olds make up 39% of the population that is 25 y and older and account for 51% of the national displaced employment among all individuals 25 y and older. *SI Appendix*, section 4 presents additional analyses by age group.

## Discussion

In this paper, we present a comparison of the initial economic and mortality consequences of the coronavirus pandemic across states and ages in the United States. All-cause excess mortality and displacement in the employment-to-population ratio are negatively correlated across age groups and positively correlated across states. Excess mortality is substantially more concentrated, affecting mostly only a few states and the oldest individuals, while the economic damages are more widespread.

Our finding of a significant positive relationship between excess all-cause mortality and economic damages across states is consistent with Chetty et al. (20), who show that counties with higher COVID case rates experienced larger declines in consumer spending in the first 2 wk of April 2020. They contrast with Rojas et al. (21), who find no relationship between increases in unemployment insurance claims and COVID case rates in March 2020, when the impact of the pandemic on both mortality and the economy was still nascent. Our findings of differences in mortality across age groups are consistent with estimates of COVID deaths by age in the United States (1, 22), in Italy (23), and in South Korea (24), which find that COVID deaths were concentrated among the elderly. Our findings of excess all-cause mortality nationally and across states are consistent with Woolf et al. (3) and Weinberger et al. (4); our findings of differences in economic outcomes by age are consistent with Bui et al. (25).

Our findings underscore the importance of examining all-cause mortality rather than just mortality labeled as a “COVID death.” COVID deaths account for only 75% of excess all-cause mortality nationally; these results are similar in magnitude to previous comparisons of national excess all-cause mortality and COVID deaths in March and April 2020 (3, 4). Also, while estimates of excess all-cause mortality by state are positively correlated with reported COVID deaths by state, the difference between the excess all-cause death rate and the COVID death rate is particularly large in states such as New York and New Jersey with the highest official COVID death rates (*SI Appendix*, section 2.3).

Some of the differences between excess all-cause mortality and COVID mortality may reflect choices in what is labeled a “COVID death,” which may include both overcounting and undercounting. However, the differences may also reflect more than just measurement: Our emphasis on all-cause mortality also highlights that the relationship between economic consequences and excess mortality may include both direct and indirect effects of the COVID pandemic. For example, the national increases in deaths due to heart disease may reflect the results of care foregone during the pandemic; this is consistent with evidence that individuals may be foregoing care for their ongoing chronic conditions or delaying care even for acute episodes of myocardial infarction or strokes (11, 26). The national decline in unnatural deaths may reflect decreased traffic fatalities due to reduced travel (14), and the decline in cancer deaths are potentially due to “harvesting” effects in which individuals who are already very ill may be more susceptible to dying from COVID.

A broader, non-COVID-specific literature has investigated the impact of changes in economic activity patterns on mortality (16–19), highlighting a variety of potential channels leading to mortality effects of varying sign. It is possible that the pandemic-induced economic recession (27) may have direct mortality impacts. For example, consistent with Ruhm (16), we find that states with higher economic damage are more likely to have reduced mortality from unnatural causes (like traffic fatalities), although the relationship is not statistically significant (*SI Appendix*, Fig. S8). Further work on the impact of the economic changes on mortality is needed.

We note several important limitations to our results. First, they reflect the situation during a specific historical moment of the initial stage of the COVID pandemic. As the pandemic progresses it is likely that the disease’s burden will be felt in different geographic areas, and the relationship between the economic burden and mortality burden may shift as consumer behaviors and employment conditions adapt. Second, while mortality is obviously an important health outcome, it is not the only one, and the pandemic may have other important impacts on morbidity that we do not capture. Third, because our analyses rely exclusively on data that are currently publicly available, we can only analyze impacts for a limited set of groups. Perhaps most importantly, we are unable to examine impacts by race; this is a very important area for further work, as existing evidence suggests that COVID deaths are disproportionately concentrated among African-Americans and Hispanics (28–30).

Nevertheless, our results highlight that health crises concentrated in one part of the country and one age group may have substantial economic spillovers that are felt throughout the rest of the country and on other age groups. In the case of age groups, the variation in the incidence of the crises on lives and livelihood may reflect natural differences in health and rates of economic activity by age. In the case of geographic variation, another mechanism may be at play—if successful at preventing the spread of the pandemic, preemptive reduction in economic activity in states not yet experiencing the health effects may also explain the combination of concentrated deaths and dispersed economic damages that we observe in the data. Overall, our results raise the possibility that the combination of concentrated health effects and diffuse economic damage may create challenges for state-level public health mitigation measures.

## Materials and Methods

### Mortality Data and Variable Construction.

All data are publicly available. National population estimates by month (2011 to 2020) come from the US Census Bureau Table NA-EST2019-01; these 2019 vintage population estimates are the latest publicly available population estimates that incorporate the latest administrative record data and methodology (31). State population estimates by year (2011 to 2019) come from the US Census Bureau Table NST-EST2019-01. We use a linear extrapolation within each state to separately estimate each state’s population in 2020. Specifically, we separately for each state estimate the following regression, where $P_{t}$ is population in year *t*: $P_{t}=\alpha_{0}+\alpha_{1}t+\epsilon_{t}$ for years t∈{2011,…,2019}. A state’s population in 2020 is then defined as ${\hat{P}}_{2020}={\hat{\alpha }}_{0}+{\hat{\alpha }}_{1}*2020$. Population estimates by age and year (2011 to 2019) come from the US Census Bureau Table NC-EST2019-AGESEX-RES; for each year, these estimates are then aggregated into our age groups. Within each age group, we estimate its 2020 population using the same linear extrapolation procedure that we used to estimate 2020 state populations.

We use weekly data on all-cause deaths by state between 2019 to 2020 from the Centers for Disease Control and Prevention’s (CDC’s) National Center for Health Statistics (NCHS) accessed on 1 July 2020, using the CDC’s weighted death count estimate, which adjusts for potential underreporting based on the degree of underreporting from each state during previous years. Comparing the CDC’s weighted and unweighted death counts for April 2020 suggests that ∼97.5% of weighted death counts are made up of reported deaths as of 1 July 2020; all states were estimated to have at least 90% of deaths reported except for North Dakota and Connecticut. See *SI Appendix*, section 2.2 for more details. All-cause death counts for each age group between 2019 and 2020 come from national weekly totals in the NCHS’s 1 July 2020 “Weekly counts of deaths by jurisdiction and age group” file and again use the CDC’s adjustment for historical underreporting. We use the finest age groupings that are available from the CDC for all-cause death counts for all years of our data. We omit the youngest age group (0 to 24 y), as the employment-to-population ratio is not defined for individuals younger than 16 y old in the CPS survey (employment questions are only administered to individuals who are 16 y and older). For 2019 and 2020, we construct monthly death counts for each state and age group by aggregating weekly death counts, assuming the one-seventh of deaths in a week take place during each day of the week. For all-cause death counts by state and age group from 2011 to 2018, we use death counts from the CDC Wonder Database based on death certificates that include information about state of residence and age (32). We calculate national death counts in all months as the sum of death counts in the 50 states and Washington, DC.

The health outcome that we consider is all-cause mortality per 10,000 people; we construct this for each state as the number of deaths in a state divided by the estimated population in the state, times 10,000. For state-level and age group-level estimates, we use the estimated annual population as the group’s population for each month within the year. In *SI Appendix*, section 2.1 we show that our estimates of excess all-cause mortality at the state level closely line up with CDC’s estimates.

For the comparison of all-cause excess deaths with COVID-attributed deaths, we use daily counts of COVID-19 deaths from *The New York Times*, which maintains a repository of daily confirmed and probable COVID-19 deaths by state, compiled from state and local health departments. These data are available through the website https://github.com/nytimes/covid-19-data.

### Economic Data and Variable Construction.

All data are publicly available. Monthly economic data from 2011 to 2020 come from the Integrated Public Use Microdata Series (IPUMS) Current Population Survey (CPS), which harmonizes data across months from the CPS (33). The CPS is a monthly survey that samples over 80,000 residents for each month. Weights are provided to construct nationally representative estimates, so when aggregating individual survey responses we use the “final basic person weight” variable, which is based on the inverse probability that an individual is selected into the sample and adjusts for known nonresponse issues. The main economic outcome that we consider is the employment-to-population ratio; this is defined as the percentage of the nonmilitary and noninstitutional population 16 y and older that either had full-time or part-time work during the week before the survey week. We construct this measure nationally, within each state, and within each age group; national measures use all 50 states and Washington, DC. In constructing age groups, we follow the finest age groups that are available for mortality data from the CDC, again omitting age group 0 to 24 y, as employment-to-population ratio is not defined in CPS for individuals younger than 16 y. We choose to focus on the employment-to-population ratio rather than the unemployment rate because the unemployment rate also captures the decision to search for work, while official counts of unemployment claims further depend on who qualifies for and applies for unemployment benefits. *SI Appendix*, section 1 discusses potential concerns about CPS survey reliability during the pandemic and validates our approach using both alternative economic measures in the CPS (specifically the unemployment rate and average hours worked per population) as well as economic measures taken from administrative data that are not affected by potential nonresponse bias; all measures are positively correlated across states and our qualitative findings are not driven by our measurement choice. Throughout, we treat CPS estimates as data, abstracting from any uncertainty around sampling error, because the replicate weights required to accurately construct SEs are not available for the monthly CPS and sampling error is plausibly very small relative to the employment effects we find.

### Predicted and Excess Outcomes.

Our goal is to calculate excess mortality and economic damage nationally and for each state and age group, for each month in the year 2020. For each outcome Y (either mortality or the employment-to-population ratio) we define the excess of the outcome in month m of year t as $EY_{mt}=Y_{mt}-PY_{mt}$, where $Y_{mt}$ is the observed value of the outcome and $PY_{mt}$ is the predicted value of the outcome. $Y_{mt}$ is directly observed in the data. In our main specification, $PY_{m,2020}$ is determined by estimating the following linear regression during the preperiod 2011 to 2019:

$$Y_{mt}=\beta t+\sum_{\mu =1}^{\mu =12}\tau_{\mu }Ind\left(\mu =m\right)+\epsilon_{mt},$$

where βt is a linear time trend, $\sum_{\mu =1}^{\mu =12}\tau_{\mu }Ind\left(\mu =m\right)$ is a sum of indicator variables for each calendar month to nonparametrically capture seasonal variation in the outcomes, and $\epsilon_{mt}$ is an error term. We use heteroskedasticity-robust SEs, clustering by year. The predicted value of the outcome in month m of 2020 is then $PY_{m,2020}=\hat{\beta }*2020+{\hat{\tau }}_{m}$, and the excess of the outcome is $EY_{m,2020}$. For our economic outcome (employment-to-population ratio) we define “excess decline in the employment–population ratio” as $-EY_{mt}$, so that a larger increase in economic damage corresponds to a larger decrease in the employment-to-population ratio.

In *SI Appendix*, section 3.1 we confirm that our approach for computing excess mortality and economic damages does not produce either meaningful excess mortality or economic damages when we replicate it for February 2020; we do observe emerging deviations from predictions in March, but they are less pronounced than in April 2020.

### Comparing Mortality and Economic Impacts.

To compare the mortality and economic impacts of COVID-19 across states, we regress excess mortality per 10,000 on economic damage (as measured by the excess decline in the employment-to-population ratio) in April 2020, where each observation is a state or Washington, DC. We use heteroskedasticity-robust SEs to construct confidence intervals for the slope of this line. In *SI Appendix*, section 3.2 we show that our findings are robust to alternative specifications of the predictive model.

## Supplementary Material

Supplementary File

## Data Availability

Stata data have been deposited in GitHub (https://git.io/JTvVF). All data used in the analyses are publicly available. Economic variables in our main analyses come from the IPUMS CPS (accessed on May 8, 2020) and death count variables in our main analyses come from the CDC’s NCHS (accessed on July 1, 2020) for years 2019 to 2020 and from the CDC’s Wonder Database (accessed on June 13, 2020) for years 2011 to 2018. Population counts to estimate mortality come from the US Census Bureau’s Tables NA-EST2019-01, NST-EST2019-01, and EST2019-AGESEX-RES (all accessed on June 13, 2020). Supplemental analyses from the *SI Appendix* use several other publicly available datasets: economic measures from the Opportunity Insights Economic Tracker (accessed on June 9, 2020 from https://tracktherecovery.org/), unemployment insurance claims from the US Department of Labor (accessed on June 8, 2020 from https://oui.doleta.gov/unemploy/claims.asp), data on industry shares from Table SAEMP27 of the Bureau of Economic Analysis (BEAS)’s Regional Data website (accessed on June 22, 2020 from https://apps.bea.gov/iTable/iTable.cfm?reqid=70&step=1&isuri=1), and counts of COVID-19 deaths from the repository maintained by *The New York Times* (accessed on June 15, 2020 from https://github.com/nytimes/covid-19-data).

## Footnotes

*Employment displacement in Michigan is not statistically significantly larger than Massachusetts, the next-largest state in terms of the employment displacement point estimates. We choose to focus on the two states with the large point estimates for consistency with the excess mortality outcome.

## References

[1] CDC, Weekly updates by select demographic and geographic characteristics. https://www.cdc.gov/nchs/nvss/vsrr/covid_weekly/index.htm. Accessed 3 August 2020.

[2] CDC COVID-19 Response Team, Geographic differences in COVID-19 cases, deaths, and incidence–United States, February 12-April 7, 2020. MMWR Morb. Mortal. Wkly. Rep. 69, 465–471 (2020). 32298250 10.15585/mmwr.mm6915e4 PMC7755058

[3] Woolf S. H., Chapman D. A., Sabo R. T., Weinberger D. M., Hill L., Excess deaths from COVID-19 and other causes, March-April 2020. JAMA 324, 510–513 (2020). 32609307 10.1001/jama.2020.11787 PMC7330820

[4] Weinberger D. M., Estimation of excess deaths associated with the COVID-19 pandemic in the United States, March to May 2020. JAMA Intern Med, e203391 (2020). 10.1001/jamainternmed.2020.3391 PMC7330834 32609310

[5] Katz J., Lu D., Sanger-Katz M., Tracking the real coronavirus death toll in the United States. *NY Times*, 23 September 2020. https://www.nytimes.com/interactive/2020/05/05/us/coronavirus-death-toll-us.html. Accessed 3 August 2020.

[6] Lu D., There has been an increase in other causes of deaths, not just coronavirus. *NY Times*, 1 June 2020. https://www.nytimes.com/interactive/2020/06/01/us/coronavirus-deaths-new-york-new-jersey.html. Accessed 3 August 2020.

[7] Coibion O., Gorodnichenko Y., Weber M., “Labor markets during the COVID-19 crisis: A preliminary view” (NBER Working Paper No. 27017, National Bureau of Economic Research, Cambridge, MA, 2020).

[8] Coibion O., Gorodnichenko Y., Weber M., “The cost of the Covid-19 crisis: Lockdowns, macroeconomic expectations, and consumer spending” (NBER Working Paper No. 27141, National Bureau of Economic Research, Cambridge, MA, 2020).

[9] Guerrieri V., Lorenzoni G., Straub L., Werning I., Macroeconomic implications of COVID-19: Can negative supply shocks cause demand shortages? SSN Electron. J., 10.3386/w26918 (2020).

[10] Baker S. R., The unprecedented stock market reaction to COVID-19. Rev. Asset Pricing Stud., raaa008 (2020).

[11] Desai S. M., Guyette F. X., Martin-Gill C., Jadhav A. P., Collateral damage–Impact of a pandemic on stroke emergency services. J. Stroke Cerebrovasc. Dis. 29, 104988 (2020). 32689650 10.1016/j.jstrokecerebrovasdis.2020.104988 PMC7284271

[12] Rosenbaum L., The untold toll–The pandemic’s effects on patients without Covid-19. N. Engl. J. Med. 382, 2368–2371 (2020). 32302076 10.1056/NEJMms2009984

[13] Kaiser Health News, Cancer patients face treatment delays and uncertainty as coronavirus cripples hospitals. https://khn.org/news/cancer-patients-face-treatment-delays-and-uncertainty-as-coronavirus-cripples-hospitals/. Accessed 3 August 2020.

[14] Brodeur A., Cook N., Wright T., “On the effects of COVID-19 safer-at-home policies on social distancing, car crashes and pollution” (IZA Discussion Paper No. 13255, IZA, Deutsche Post Foundation, 2020).

[15] National Safety Council, Preliminary monthly estimates. Injury facts. https://injuryfacts.nsc.org/motor-vehicle/overview/preliminary-monthly-estimates/. Accessed 3 August 2020.

[16] Ruhm C. J., Are recessions good for your health? Q. J. Econ. 115, 617–650 (2000).

[17] Ruhm C. J., Healthy living in hard times. J. Health Econ. 24, 341–363 (2005). 15721049 10.1016/j.jhealeco.2004.09.007

[18] Ruhm C. J., Recessions, healthy no more? J. Health Econ. 42, 17–28 (2015). 25839783 10.1016/j.jhealeco.2015.03.004

[19] Cutler D., Deaton A., Lleras-Muney A., The determinants of mortality. J. Econ. Perspect. 20, 97–120 (2006).

[20] Chetty R., Friedman J. N., Hendren N., Stepner M., “How did COVID-19 and stabilization policies affect spending and employment? A new real-time economic tracker based on private sector data” (NBER Working Paper No. 27431, National Bureau of Economic Research, Cambridge, MA, 2020).

[21] Rojas F. L., “Is the cure worse than the problem itself? Immediate labor market effects of COVID-19 case rates and school closures in the U.S.” (NBER Working Paper No. 27127, National Bureau of Economic Research, Cambridge, MA, 2020).

[22] Knittel C. R., Ozaltun B., What does and does not correlate with COVID-19 death rates. medRxiv:2020.06.09.20126805 (11 June 2020).

[23] Modi C., Total COVID-19 mortality in Italy: Excess mortality and age dependence through time-series analysis. medRxiv:2020.04.15.20067074 (20 April 2020).

[24] Dowd J. B., Demographic science aids in understanding the spread and fatality rates of COVID-19. Proc. Natl. Acad. Sci. U.S.A. 117, 9696–9698 (2020). 32300018 10.1073/pnas.2004911117 PMC7211934

[25] Bui T. T. M., Button P., Picciotti E. G., “Early evidence on the impact of COVID-19 and the recession on older workers” (NBER Working Paper No. 27448, National Bureau of Economic Research, Cambridge, MA, 020).

[26] Garcia S., Reduction in ST-segment elevation cardiac catheterization laboratory activations in the United States during COVID-19 pandemic. J. Am. Coll. Cardiol. 75, 2871–2872 (2020). 32283124 10.1016/j.jacc.2020.04.011 PMC7151384

[27] NBER, Determination of the February 2020 peak in US economic activity. https://www.nber.org/cycles/june2020.html. Accessed 3 August 2020.

[28] Ford T., Reber S., Reeves R. V., Race gaps in COVID-19 deaths are even bigger than they appear. *Brookings* (2020). https://www.brookings.edu/blog/up-front/2020/06/16/race-gaps-in-covid-19-deaths-are-even-bigger-than-they-appear/. Accessed 3 August 2020.

[29] McLaren J., “Racial disparity in COVID-19 deaths: Seeking economic roots with Census data” (NBER Working Paper No. 27407, National Bureau of Economic Research, Cambridge, MA, 2020).

[30] Price-Haywood E. G., Burton J., Fort D., Seoane L., Hospitalization and mortality among black patients and white patients with COVID-19. N. Engl. J. Med. 382, 2534–2543 (2020). 32459916 10.1056/NEJMsa2011686 PMC7269015

[31] US Census Bureau 2019 National and State Population Estimates. https://www.census.gov/newsroom/press-kits/2019/national-state-estimates.html. Accessed 13 June 2020.

[32] Multiple Cause of Death Data CDC Wonder Database. https://wonder.cdc.gov/. Accessed 13 June 2020.

[33] Flood S., King M., Rodgers R., Ruggles S., Warren J. R., Integrated Public Use Microdata Series, Current Population Survey: Version 8.0. https://cps.ipums.org/cps/. Accessed 8 May 2020.

