# Supplementary material for: Initial economic damage from the COVID-19 pandemic in the United States is more widespread across ages and geographies than initial mortality impacts
Source: Proc Natl Acad Sci U S A. 2020 Oct 20;117(45):27934–9. doi: 10.1073/pnas.2014279117 (PMC7668078; doi:10.1073/pnas.2014279117)
Supplement: Supplementary File [file pnas.2014279117.sapp.pdf]

Supplementary Information for:

Initial economic damage from the COVID-19 pandemic in the U.S. is more widespread across ages and geographies than initial mortality impacts.

AUTHORS: Maria Polyakova; Geoffrey Kocks; Victoria Udalova; Amy Finkelstein

To whom correspondence should be addressed. E-mail: afink@mit.edu

This PDF file includes:

- Supplementary text
- Figures S.1 to S.11
- Tables S.1 to S.9

1 Validation of Economic Damage Measure

1.1 CPS Response Rates During COVID-19 Pandemic

CPS randomly samples households in the United States, not including institutionalized populations. One person in the household answers for all members. Households are surveyed for 4 consecutive months, left out for 8 consecutive months, and surveyed for the following 4 months. Approximately 54,000 households (105,000 individuals) end up being sampled each month.[1]

During the COVID-19 pandemic, the Bureau of Labor Statistics (BLS) reports the following effects on the survey (report for March 2020: https://www.bls.gov/cps/employment-situation-covid19-faq-march-2020.pdf and April 2020: https://www.bls.gov/cps/employment-situation-covid19-faq-april-2020.pdf). In March and April 2020, response rates were lower (73% and 70% vs. a typical average of 83%) than usual due to the closure of call centers and the inability to do in-person visits. BLS notes that some workers may have been misclassified as "employed but not at work" rather than "unemployed on temporary layoff."

The lower response rate during the pandemic raises some concerns about using the CPS to estimate differential economic damages across groups, especially since previous research suggests that the "missing" respondents in the March 2020 sample were not a random sample of CPS respondents, being somewhat younger, more racially diverse, and having lower educational attainment (1). BLS suggests, however, that overall "although the response rate was adversely affected by pandemic-related issues, BLS was still able to obtain estimates that met our standards for accuracy and reliability" and prior researchers have used the April 2020 CPS to study the economic impacts of the pandemic (2). However, due to concerns of non-response bias, we validate our CPS measure of economic damages by state against other measures based on administrative data from the Opportunity Insights Economic Tracker and unemployment insurance data from the BLS; as described in Section 1.2 and 1.3 below, the results are reassuring: all measures are positively correlated across states and our qualitative findings are robust to alternative measurement choices. An advantage of the CPS data over these measures is that it allows us to examine economic damage by demographic cuts such as age.

1.2 Comparison to Other Economic Measures

As our economic damage measure, we use the difference between the observed and expected employment-population ratio from the April 2020 CPS. Table S.1 below confirms that across states, this measure is correlated with several other measures of economic damage:

- **Excess unemployment rate** within each state is based on the unemployment rate from the April 2020 CPS and uses a linear trend since 2011 with month fixed effects to estimate the expected unemployment rate. The unemployment rate is defined as the percentage of the labor force that was not employed and have looked for work in the past four

[1] Source: https://www.bls.gov/opub/hom/cps/design.htm

www.pnas.org/cgi/doi/10.1073/pnas.2014279117

weeks, where the labor force is non-military and non-institutionalized individuals 16 and older who are either working or looking for work.

- The insured unemployment rate is calculated - separately from the CPS data - by the Department of Labor as the number of continued claims divided by the number of covered employees (individuals who would be eligible for unemployment insurance), where continued claims refer to individuals who had already filed initial claims during the previous week. For each month, we determine the excess insured unemployment rate for the week after the CPS's reference week for that month. The predicted insured unemployment rate in April 2020 is then estimated within each state using a linear trend since 2011 with month fixed effects and the **excess insured unemployment rate** is the difference between the observed rate and the predicted rate.

- The percent of the population with unemployment insurance (UI) claims in each state is 100 times the number of continued claims divided by the estimated population. The **excess percent of the population with UI claims** is estimated using the same procedure as above.

- The percent **decrease in consumer spending** between January 4-31, 2020 and April 15, 2020 comes from the Opportunity Insights Economic Tracker (https://tracktherecovery.org/) and is calculated by using credit and debit card data from Affinity Solutions. Spending for each date is calculated using a seasonally adjusted seven-day moving average. The percent change since January 2020 for each day is calculated by dividing that day's seasonally adjusted moving average by the mean between January 4-31, 2020, and subtracting this value from 1.

- The percent **decrease in small business employment** between January 4-31, 2020 and April 15, 2020 also comes from the Opportunity Insights Economic Tracker and is calculated using data from Homebase, which primarily includes food, retail, and services. Small business employment is calculated using a seven-day moving average. The percent change since January 4-31, 2020 for each day is calculated by dividing that day's moving average by the mean in January 4-31, 2020, and subtracting this value from 1.

Table S.1: Economic Measures Correlation Table - April 2020

	(1) Excess Decline in Employment-Population Ratio	(2) Excess Unemployment Rate	(3) Excess Insured Unemployment Rate	(4) Excess Percent of Pop. with UI Claims	(5) Percent Decrease in Consumer Spending	(6) Percent Decrease in Small-Business Employment
(1) Excess Decline in Employment-Population Ratio	1.000	0.863	0.402	0.409	0.199	0.620
(2) Excess Unemployment Rate	.	1.000	0.433	0.405	0.093	0.484
(3) Excess Insured Unemployment Rate	.	.	1.000	0.952	0.220	0.598
(4) Excess Percent of Pop. with Unemployment Insurance Claims	.	.	.	1.000	0.288	0.659
(5) Percent Decrease in Consumer Spending	.	.	.	.	1.000	0.232

Notes: This table shows the correlations between each of the indicated economic measures across the 50 states and Washington, D.C. in April 2020, with each measure defined in Section 1.2.

1.3 Robustness of Results to Other Economic Measures

In Table S.2, we show that the positive relationship between excess mortality and economic damage is robust to alternate economic measures.

Row (1) shows the baseline results from the employment-population ratio.

Row (2) uses a *modified employment-population ratio* from the CPS data which takes into account the CPS's observation in April 2020 that there were likely many workers in the survey who were misclassified as "employed but absent from work" when they actually should have been classified as unemployed or on a temporary layoff.[2] Since the CPS does not adjust these responses, to take into account potential misclassification, we construct a the modified employment-population ratio measure; this is defined as the percentage of the non-military and non-institutional population 16 and older that either had full-time or part-time work during the week before the survey week, excluding individuals who were absent from work for an unlisted reason.[3]

Row (3) uses "average hours per population" from the CPS data, where "average hours per population" is defined as the total number of hours worked divided by the total non-military and non-institutionalized population 16 years and older.

Row (4) uses the unemployment rate from the CPS data, as defined in the previous section.

Row (5) uses the insured unemployment rate from the BLS, as defined in the previous section.

Each measure points to substantial national economic damage in April 2020. In addition, when using the modified employment-population ratio and average hours per population, the slope from regressing excess mortality on economic damage remains positive and significant at the 5% level, both among all states and when excluding the two states with the largest excess mortality - New York and New Jersey. The slope remains positive but is no longer significant when using the unemployment rate or the insured unemployment rate.

Table S.2: Robustness: Alternate Economic Measures (April 2020)

Outcome	(1) Observed	(2) Predicted	(3) Deviation from Predicted	(4) Slope from Regressing Excess Mortality (per 10,000) on Economic Damage	(5) Slope from Regressing Excess Mortality (per 10,000 on Economic Damage (Excluding NY and NJ)
(1) Employment-Population Ratio	51.4	61.3<br>[61.1, 61.4]	9.9<br>[9.7, 10.0]	0.220<br>[0.045, 0.395]	0.168<br>[0.022, 0.313]
(2) Modified Employment-Population Ratio	48.3	61.1<br>[60.9, 61.2]	12.8<br>[12.6, 12.9]	0.285<br>[0.093, 0.477]	0.187<br>[0.063, 0.312]
(3) Average Hours per Population	17.7	23.2<br>[23.0, 23.4]	5.5<br>[5.4, 5.7]	0.544<br>[0.143, 0.944]	0.419<br>[0.093, 0.744]
(4) Unemployment Rate	14.5	2.2<br>[1.5, 2.9]	12.3<br>[11.6, 13.0]	0.115<br>[-0.052, 0.282]	0.053<br>[-0.068, 0.174]
(5) Insured Unemployment Rate	14.9	0.8<br>[0.5, 1.0]	14.1<br>[13.9, 14.4]	0.160<br>[-0.005, 0.325]	0.082<br>[-0.023, 0.187]

Notes: This table shows the robustness of results to various economic measures. For each economic outcome, column (1) shows the observed national value of the outcome in April 2020 and column (2) shows the predicted value of the outcome in April 2020 based on a linear time trend from 2011-2019 with month fixed effects. Column (3) shows the resulting deviation from the prediction, where a larger deviation corresponds to more economic damage. Column (4) uses excess mortality and economic damage in April 2020 calculated separately by state and reports the slope from regressing excess mortality on economic damage; regressions give each state equal weight and heteroskedasticity-robust standard errors are used for calculating confidence intervals. Column (5) shows robustness to excluding New York and New Jersey from the regression from Column (4). 95% confidence intervals are shown underneath each estimate in Columns (2)-(5).

[2] See Section 13 of the April 2020 FAQ from the CPS: https://www.bls.gov/cps/employment-situation-covid19-faq-april-2020.pdf.

[3] Individuals who were employed but absent from work are asked by the CPS why they were absent from work, and possible options are vacation and personal days, illnesses or medical problems, child care problems, personal or family obligations, maternity/paternity leave, labor disputes, weather, school, and military duty. Other reasons are "unlisted reasons."

2 Validation of Excess Mortality Measure

2.1 Comparison to CDC Excess Mortality

At the state level, we are able to assess how well our excess mortality measure lines up with existing estimates by benchmarking our estimates against the difference between "observed mortality" and "average expected mortality" from the CDC's "Excess Deaths Associated with COVID-19" file from the NCHS (using the July 1, 2020 update).[4]

In Figure S.1, we plot our estimates of the excess mortality rate in each state in April 2020 against the difference between observed mortality and average expected mortality from the CDC; we calculate their observed mortality as their "observed number" divided by state population estimates from the U.S. Census Bureau, with an analogous procedure for average expected mortality. This shows that our excess mortality estimates at the state level line up closely with the CDC's estimated differences between observed and predicted mortality, despite the use of a different statistical methodology for estimating predicted mortality.

[4]We are only able to perform this exercise by state rather than age group, because average expected deaths are not available from the CDC by age group.

Figure S.1: Excess Mortality Comparisons by State

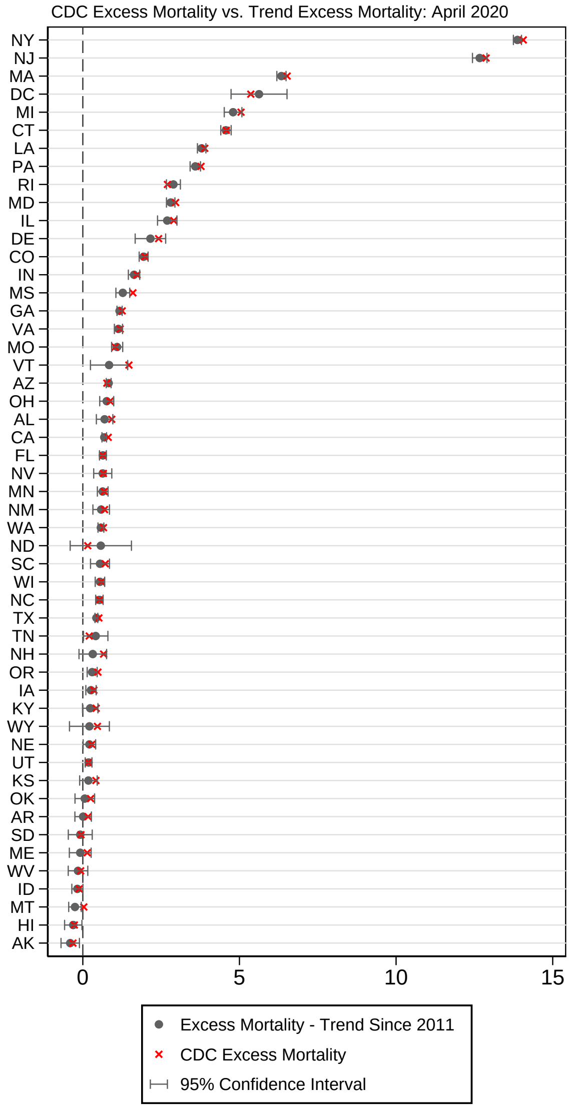

Notes: This figure plots our estimates of excess mortality by state in April 2020, using a linear time trend since 2011 with month fixed effects, and the associated confidence intervals. The red X marks indicate the difference between observed mortality and average expected mortality by state from the CDC.

2.2 Underreporting of Recent Deaths

In their data of death counts, the CDC note that data for recent weeks may be incomplete based on lags in the reporting of deaths by state. However, both an unweighted death count, which does not make any adjustments for underreporting, and a weighted death count, which adjusts death counts based on historical levels of underreporting, are provided. For all of our mortality analyses, we use weighted death counts as our outcome of interest. However, by comparing unweighted to weighted death counts by states, we can gauge the extent to which our data are complete rather than estimated.

We focus on April 2020 in our analyses because data are largely complete for April by the time of our analysis (July 1, 2020). Based on our calculations of differences between their reported "observed deaths" and "average expected deaths," the CDC estimated that 97.5% of April deaths and 95% of May deaths were reported by July 1. However, completeness varies by state; by July 1, only two states (North Dakota and Connecticut) were estimated to have less than 90% of deaths reported in April 2020, while nine states were estimated to have less than 90% of deaths reported in May 2020. We therefore focus our analysis on April 2020. Other, earlier papers, have analyzed the data from March through May (3, 4).

Table S.3 confirms that our results are not sensitive to potential underreporting of recent deaths or adjustments by the CDC of these deaths. Row (1) shows results using our baseline mortality variable of death counts adjusted by the CDC to account for underreporting, while row (2) uses unadjusted death counts. In addition, in Column (5), slopes from regressing excess mortality on economic damage exclude North Dakota and Connecticut, to confirm that our results are not driven by states with low reporting rates. In all cases, there is a substantial national increase in mortality in April 2020, and the slope from regressing excess mortality and economic damage is always positive and significant.

Table S.3: Robustness: Alternate Death Measures (April 2020)

Death Measure	(1) Observed Mortality (per 10,000)	(2) Predicted Mortality (per 10,000)	(3) Deviation from Prediction	(4) Slope from Regressing Excess Mortality (per 10,000) on Economic Damage	(5) Slope from Regressing Excess Mortality (per 10,000) on Economic Damage, Excluding Low-Reporting States
(1) Baseline: Adjusted Deaths	9.7	7.3<br>[7.2, 7.4]	2.4<br>[2.3, 2.4]	0.220<br>[0.045, 0.395]	0.225<br>[0.049, 0.402]
(2) Unadjusted Deaths	9.5	7.3<br>[7.2, 7.4]	2.1<br>[2.1, 2.2]	0.233<br>[0.061, 0.405]	0.218<br>[0.048, 0.387]

Notes: This table shows the robustness of results to using unadjusted deaths rather than adjusted deaths, and to excluding low-reporting states. For each mortality outcome, column (1) shows the observed national value of the outcome in April 2020 and column (2) shows the predicted value of the outcome in April 2020 based on a linear time trend from 2011-2019 with month fixed effects. Column (3) shows the resulting deviation from the prediction. The regression in Column (4) gives each state equal weight and heteroskedasticity-robust standard errors are used for calculating confidence intervals. Column (5) excludes North Dakota and Connecticut - the two states with less than 90% of April 2020 deaths reported as of July 1, 2020. 95% confidence intervals are shown for each estimate in Columns (2)-(5).

2.3 Comparison to COVID-19 Mortality

As discussed in the main text, excess mortality may not line up with reported COVID-19 mortality for a number of reasons, including limited testing and simultaneous changes in mortality from other causes of death. In Figure S.2, we compare our excess mortality measure in each state to recorded COVID-19 deaths in April 2020 (from the repository from *The New York Times*) divided by the estimated population from the U.S. Census Bureau, among the 49 states where the CDC estimates that at least 90% of all-cause deaths were reported by July 1. While excess mortality is positively correlated with COVID-19 mortality, excess mortality is much higher than COVID-19 mortality for some of the most affected states, such as New York and New Jersey.

Table S.4 shows, for the United States and for the top five states by excess all-cause mortality, the estimated number of COVID-19 deaths, the estimated number of excess deaths, and COVID-19 deaths as a share of excess deaths. Nationally, approximately 75% of estimated excess deaths are accounted for by COVID-19 deaths; among the top five states, this ranges from 53% in Washington, D.C. to 80% in New York.

Figure S.2: Comparisons of COVID and Excess Mortality

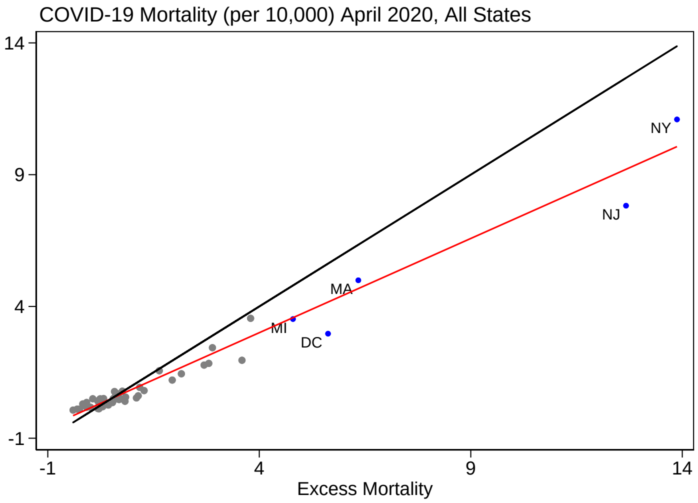

Notes: This figure plots COVID-19 mortality (per 10,000) in April 2020 against estimated all-cause excess mortality (per 10,000) in April 2020, where each observation is one state or Washington, D.C. The black line shows the 45-degree line and the red line shows the line of best fit.

Table S.4: COVID-19 Deaths and Excess Deaths Comparison (April 2020)

	(1) Estimated COVID-19 Deaths	(2) Estimated Excess Deaths	(3) COVID-19 Deaths as Share of Excess Deaths
United States	58,745	77,514	75.79%
**Individual States**			
NY	21,687	27,119	79.97%
NJ	6,961	11,275	61.74%
MA	3,473	4,411	78.74%
DC	215	408	52.73%
MI	3,524	4,800	73.42%

Notes: This table compares estimated COVID-19 deaths to estimated excess deaths. Column (1) shows COVID-19 deaths, nationally and by state in April 2020, from *The New York Times*, using both confirmed and probable COVID deaths. Column (2) shows estimated excess deaths, estimating excess mortality with a linear time trend since 2011 with month fixed effects and multiplying this excess mortality by population estimates from the Census. Column (3) shows COVID-19 deaths as a share of excess deaths.

3 Validation of Model Specification

3.1 Outcomes in February and March

As a check that our model for predicted mortality and economic outcomes is reasonable, we would expect predictions prior to COVID-19 to be close to observed outcomes. In Figure S.3, we confirm that our national predictions of each outcome line up closely with the true values in February 2020; observed values begin diverging in March 2020 but are not nearly as severe as in April.

Figure S.3: Time Series Within Alternate February and March

(a) Mortality (per 10,000)

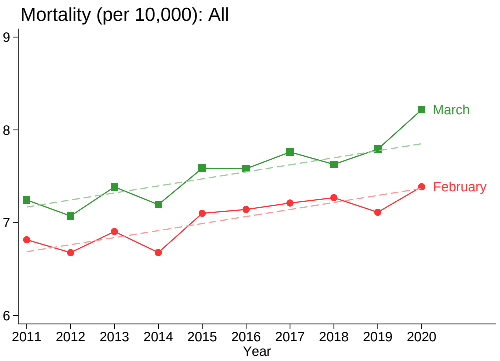

(b) Employment-Population Ratio

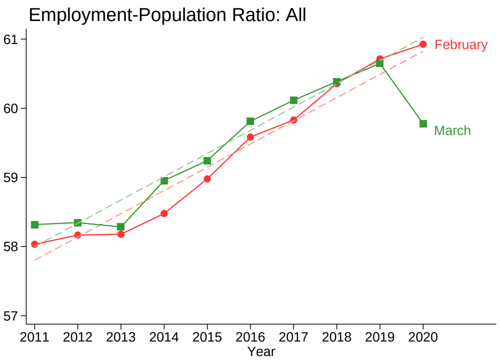

Notes: These figures show time series of (a) mortality per 10,000 and (b) employment-population ratio for each February and March from 2011-2020. Solid lines show observed values in each month, while lighter dashed lines show the values predicted based on the linear time trend from 2011-2019 with month fixed effects.

3.2 Robustness to Alternate Specifications

In Table S.5, we confirm that our results are robust to changes in how we model predictions for each outcome. Our baseline specification uses a linear trend from 2011-2019 with month fixed effects. Results remain similar when using a linear trend from 2015-2019 or when predicting each outcome in April 2020 as its average in each April from 2015-2019.

Table S.5: Robustness: Alternate Prediction Specifications

	**Specification**		
Outcome	Baseline (Linear Trend Since 2011)	Linear Trend Since 2015	Within-Month Average Since 2015
(1) Predicted Mortality (per 10,000)	7.3<br>[7.2, 7.4]	7.3<br>[7.2, 7.4]	7.1<br>[7.0, 7.2]
(2) Deviation from Predicted Mortality	2.4<br>[2.3, 2.4]	2.4<br>[2.3, 2.5]	2.6<br>[2.5, 2.7]
(3) Predicted Employment-Population Ratio	61.3<br>[61.1, 61.4]	61.5<br>[61.1, 61.8]	60.3<br>[59.7, 60.9]
(4) Deviation from Predicted Employment-Population Ratio	9.9<br>[9.7, 10.0]	10.1<br>[9.7, 10.4]	8.9<br>[8.3, 9.5]
(5) Slope from Regressing Excess Mortality on Economic Damage	0.220<br>[0.045, 0.395]	0.205<br>[0.048, 0.362]	0.240<br>[0.042, 0.438]

Notes: This table shows the robustness of results to various specifications used to predict excess mortality and economic damage in April 2020. The baseline specification uses a linear trend from 2011-2019 with month fixed effects. The first alternate specification uses a linear trend from 2015-2019 with month fixed effects. The second alternate specification uses the within-month average from 2015-2019. For each specification, row (1) shows the resulting predicted national mortality per 10,000 and row (2) shows the difference between observed mortality per 10,000 and predicted mortality per 10,000. Row (3) shows the resulting predicted employment-population ratio and row (4) shows the difference between the observed employment-population ratio and the predicted employment-population ratio, where a larger deviation corresponds to higher levels of economic damage. Row (5) estimates the predicted mortality and predicted employment-population ratio separately within each state and then reports the slope from regressing excess mortality on economic damage, where each state is given an equal weight and heteroskedasticity-robust standard errors are used for calculating confidence intervals. 95% confidence intervals are shown under each estimate.

4 Results Within Age Groups

To provide full results by age group available from the CDC, Table S.6 shows economic and mortality results both overall and by age group in April 2020. Figures S.4 and S.5 show predictions and observed values in April of each year for mortality

and the employment-population ratio. These show that for all age groups and for each outcome, the trends fit well in each April prior to 2020.

Table S.6: Heterogeneity in Outcomes by Age Group: April 2020

Group	(1) Observed	(2) Predicted	(3) Deviation from Predicted	(4) % Deviation from Predicted
**Employment-Population Ratio**				
All Age Groups (25+)	54.2	63.0<br>[62.8, 63.2]	8.8<br>[8.6, 9.0]	14.0%<br>[13.7%, 14.3%]
Ages 25-44	69.5	81.1<br>[80.9, 81.4]	11.6<br>[11.3, 11.9]	14.3%<br>[14.0%, 14.6%]
Ages 45-64	63.0	72.0<br>[71.6, 72.3]	9.0<br>[8.7, 9.4]	12.6%<br>[12.1%, 13.0%]
Ages 65-74	22.8	27.8<br>[27.4, 28.2]	5.0<br>[4.6, 5.4]	17.9%<br>[16.7%, 19.1%]
Ages 75-84	8.6	10.9<br>[10.6, 11.2]	2.3<br>[2.0, 2.6]	21.0%<br>[18.9%, 23.1%]
Ages 85+	3.5	5.3<br>[4.7, 5.9]	1.8<br>[1.2, 2.4]	33.7%<br>[26.1%, 41.3%]
**Mortality (per 10,000)**				
All Ages Groups (25+)	13.8	10.4<br>[10.3, 10.5]	3.4<br>[3.3, 3.5]	32.8%<br>[31.6%, 34.1%]
Ages 25-44	1.7	1.4<br>[1.3, 1.4]	0.3<br>[0.3, 0.4]	22.4%<br>[18.1%, 26.7%]
Ages 45-64	6.8	5.4<br>[5.3, 5.4]	1.5<br>[1.4, 1.5]	27.0%<br>[25.1%, 28.8%]
Ages 65-74	19.1	14.5<br>[14.4, 14.7]	4.6<br>[4.4, 4.7]	31.5%<br>[30.1%, 32.9%]
Ages 75-84	49.4	35.5<br>[35.1, 35.8]	13.9<br>[13.6, 14.3]	39.2%<br>[37.8%, 40.6%]
Ages 85+	148.8	109.8<br>[108.3, 111.4]	39.0<br>[37.5, 40.6]	35.5%<br>[33.6%, 37.4%]

Notes: This table shows full economic and mortality results overall and by CDC age group. For each outcome, column (1) shows the observed national value of the outcome in April 2020 and column (2) shows the predicted value of the outcome in April 2020 based on a linear time trend from 2011-2019 with month fixed effects. Column (3) shows the resulting deviation from the prediction; a larger deviation corresponds to larger economic or mortality damages. Column (4) shows the percent deviation from the prediction, using the delta method to construct standard errors. Columns (2)-(4) show 95% confidence intervals underneath the estimates.

Figure S.4: April Mortality Time Series by Age Group

(a) Mortality per 10,000: 25-44

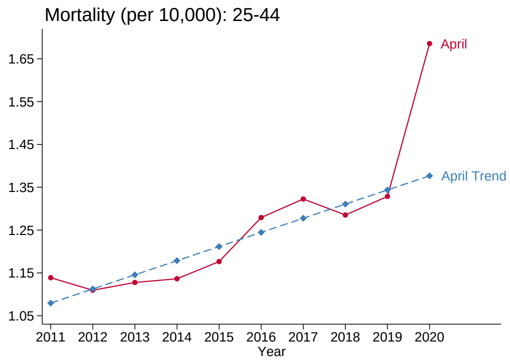

(b) Mortality per 10,000: 45-64

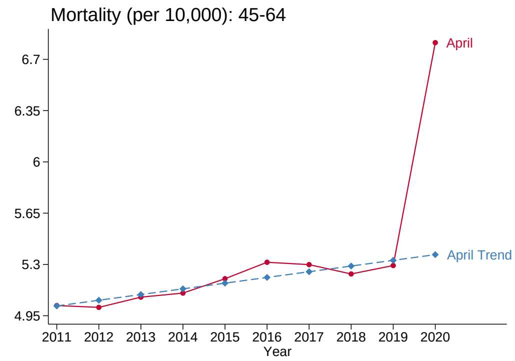

(c) Mortality per 10,000: 65-74

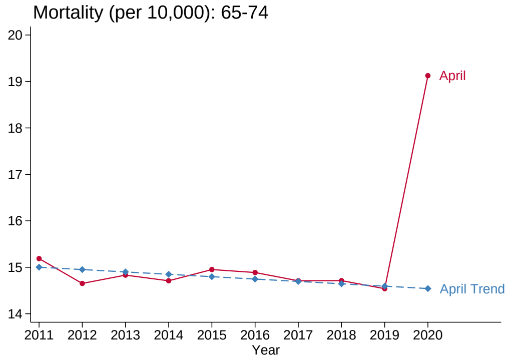

(d) Mortality per 10,000: 75-84

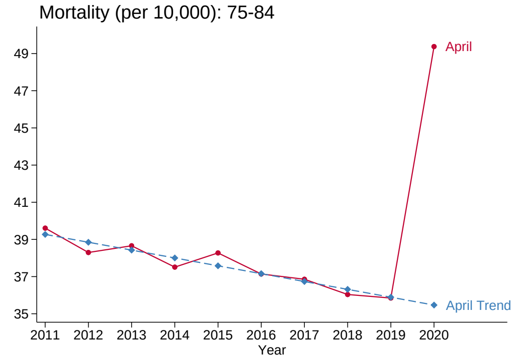

(e) Mortality per 10,000: 85+

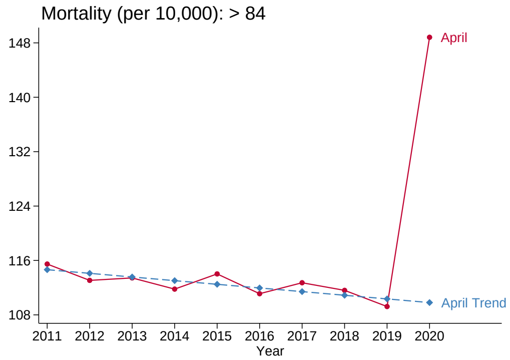

Notes: These figures show the time series for the monthly mortality rate in April of each year, where each panel is a different CDC age group. Predictions are calculated within each group using a linear time trend with month fixed effects during the period 2011-2019.

Figure S.5: April Employment-Population Ratio Time Series by Age Group

(a) Employment-Population Ratio: 25-44

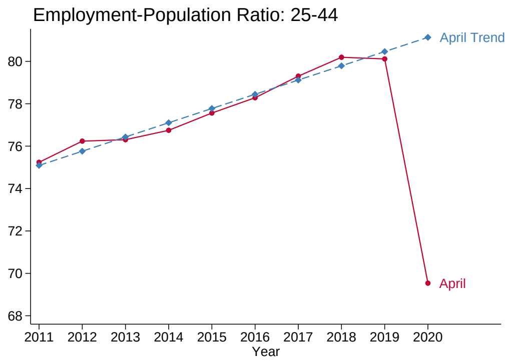

(b) Employment-Population Ratio: 45-64

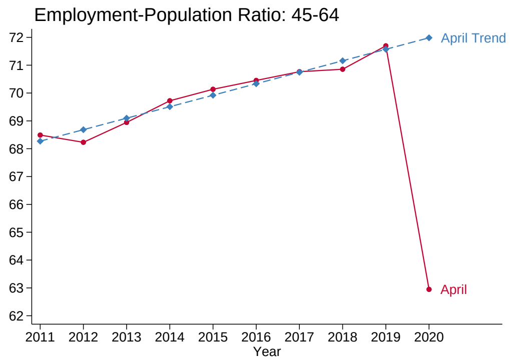

(c) Employment-Population Ratio: 65-74

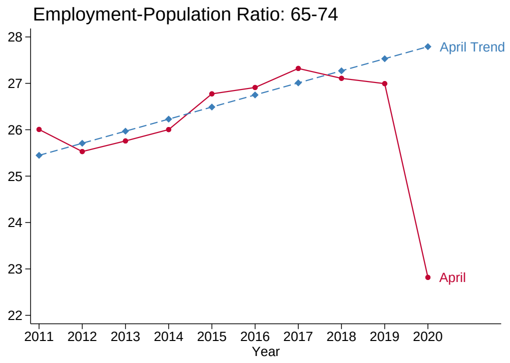

(d) Employment-Population Ratio: 75-84

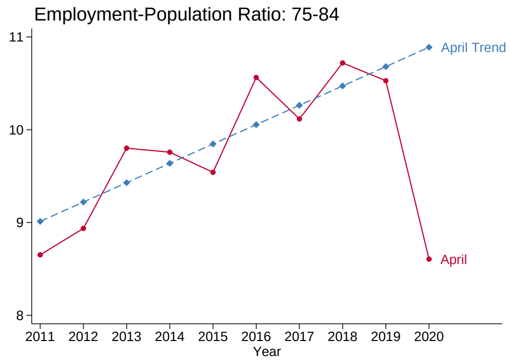

(e) Employment-Population Ratio: 85+

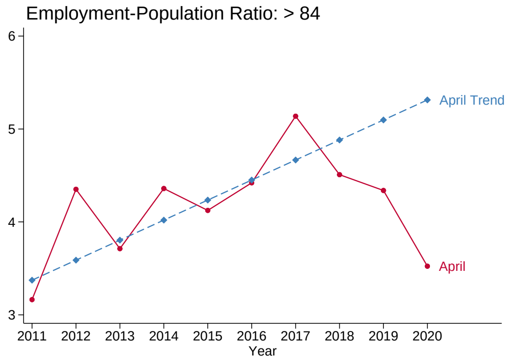

Notes: These figures show the time series for the monthly employment-population ratio in April of each year, where each panel is a different CDC age group. Predictions are calculated within each group using a linear time trend with month fixed effects during the period 2011-2019.

5 State Contributions to National Outcomes

To determine the relative contribution of damage from the hardest hit states to national damage for each outcome, Table S.7 ranks the top 5 states based on their per capita excess decline in the employment-population ratio and excess mortality. Column (1) shows the contribution of each group of states to the national excess deaths or employment displacement, using national estimates from overall national trends.

Table S.8 presents an alternate listing of the top 5 states for each outcome, based on the top 5 states as measured by displaced employment or excess deaths; this ranking takes into account the fact that states with high per capita excess outcomes may not necessarily have the largest contributions to national outcomes due to differences in population.

Table S.7: Share of Excess Damage in Top Per-Capita States

	(1) % of National Impact	(2) List of States
**Excess Decline in Emp-Pop Ratio**		
Top State	1.77%	NV
Top 2 States	7.13%	NV, MI
Top 3 States	10.81%	NV, MI, MA
Top 4 States	11.47%	NV, MI, MA, HI
Top 5 States	14.46%	NV, MI, MA, HI, IN
**Excess Mortality**		
Top State	34.65%	NY
Top 2 States	49.06%	NY, NJ
Top 3 States	54.70%	NY, NJ, MA
Top 4 States	55.22%	NY, NJ, MA, DC
Top 5 States	61.35%	NY, NJ, MA, DC, MI

Notes: This table shows the five states with the largest point estimates for economic damage and excess mortality per capita within each state; excess mortality and economic damage is computed separately within each state using a linear time trend from 2011-2019 with month fixed effects. Column (1) shows the percentage of the national economic damage or excess mortality accounted for by each of these states, where the national damage is computed for the country as a whole from 2011-2019 with month fixed effects. Column (2) lists the states in each group (including Washington, D.C.).

Table S.8: Share of Excess Damage in States with Highest Excess Deaths and Displaced Employment

	(1) % of National Impact	(2) List of States
**Displaced Employment**		
Top State	13.72%	CA
Top 2 States	21.86%	CA, TX
Top 3 States	29.49%	CA, TX, FL
Top 4 States	36.26%	CA, TX, FL, NY
Top 5 States	41.62%	CA, TX, FL, NY, MI
**Excess Deaths**		
Top State	34.65%	NY
Top 2 States	49.06%	NY, NJ
Top 3 States	55.19%	NY, NJ, MI
Top 4 States	61.07%	NY, NJ, MI, PA
Top 5 States	66.71%	NY, NJ, MI, PA, MA

Notes: This table shows the five states with the largest point estimates for displaced employment and excess deaths within each state; excess mortality and economic damage is computed separately within each state using a linear time trend from 2011-2019 with month fixed effects. Column (1) shows the percentage of the national economic damage or excess mortality accounted for by each of these states, where the national damage is computed for the country as a whole from 2011-2019 with month fixed effects. Column (2) lists the states in each group (including Washington, D.C.).

6 Comparisons of Excess Outcomes to Industry Shares

Industry shares may play a role in mitigating or exacerbating the economic consequences of the pandemic. For each state, we calculate the share of the total employed population (both full-time and part-time) that works in each of 18 industry categories

listed by the BEA in 2018, the last year of data that they have available (https://apps.bea.gov/iTable/iTable.cfm?reqid=70&step=1&isuri=1, Table SAEMP27). For each industry, Table S.9 lists the three states with the largest share of workers employed in that industry group, as well as the slopes and 95% confidence intervals from regressing excess mortality and economic damage on the state industry shares.

This illustrates that some industries - such as arts, entertainment, recreation, accommodations, and food services - are associated with more economic damage, despite no impact on excess mortality. On the other hand, other industries - such as professional, scientific, and technical services - are associated with positive excess mortality, likely reflecting the geographic distribution of the industries, but no significant impact on economic damage.

Table S.9: Economic and Mortality Damage vs. Industry Shares

		**Slope from regressing outcome on state industry share (2018):**	
BEA Industry	Top 3 States	Excess Mortality (per 10,000)	Economic Damage
Forestry and Fishing	AK, ME, ID	-2.75<br>[-4.41, -1.09]	-3.69<br>[-5.62, -1.77]
Mining, quarrying, oil, and gas	WY, OK, ND	-0.45<br>[-0.78, -0.13]	-1.01<br>[-1.38, -0.64]
Utilities	WY, ND, WV	-3.17<br>[-6.42, 0.07]	-8.17<br>[-16.14, -0.20]
Construction	LA, MT, WY	-1.30<br>[-2.29, -0.32]	-0.75<br>[-1.83, 0.33]
Manufacturing	IN, WI, MI	-0.21<br>[-0.48, 0.06]	0.11<br>[-0.32, 0.53]
Wholesale Trade	ND, NJ, IL	0.23<br>[-1.48, 1.94]	0.29<br>[-1.16, 1.74]
Retail Trade	NH, ME, WV	-0.93<br>[-1.52, -0.33]	-0.22<br>[-1.01, 0.56]
Transportation and Warehousing	NV, IL, NJ	0.25<br>[-0.71, 1.20]	0.80<br>[-0.28, 1.88]
Information	WA, CA, NY	1.98<br>[-0.51, 4.46]	0.84<br>[-0.98, 2.67]
Finance and Insurance	DE, CT, NY	0.68<br>[-0.03, 1.39]	0.19<br>[-0.34, 0.73]
Real estate, rental, and leasing	FL, NJ, WY	1.01<br>[-0.37, 2.40]	0.81<br>[-0.44, 2.05]
Professional, scientific, technical services	DC, VA, MA	0.63<br>[0.29, 0.96]	0.36<br>[-0.25, 0.96]
Company management	MN, RI, AR	0.85<br>[-0.52, 2.21]	1.80<br>[-0.21, 3.81]
Administrative and Support	AZ, GA, FL	0.15<br>[-0.27, 0.57]	1.34<br>[0.53, 2.14]
Educational Services	DC, MA, RI	1.22<br>[0.59, 1.84]	0.86<br>[-0.24, 1.96]
Health care and Social Assistance	PA, MA, NY	0.52<br>[0.01, 1.04]	0.28<br>[-0.42, 0.97]
Arts, Entertainment, and Recreation	NV, MT, NY	1.36<br>[-0.61, 3.33]	3.25<br>[1.06, 5.44]
Accommodation and food services	NV, HI, WY	-0.30<br>[-0.67, 0.07]	0.82<br>[0.46, 1.18]

Notes: This table shows the relationship between industry shares and mortality damage and economic damage, using the 18 categories listed by the BEA in 2018. The top three states are listed for each industry, based on the share of the population in those states that are estimated to work in the given industry. The column for "excess mortality" regresses excess mortality in April 2020 on industry shares, where each observation is one state and the regression is unweighted. The column for "economic damage" regresses economic damage (as measured by the excess decline in the employment-population ratio) in April 2020 on industry shares, where each observation is one state and the regression is unweighted. 95% confidence intervals are shown for each, and use heteroskedasticity-robust standard errors.

7 Results by Cause of Death

7.1 COVID Mortality and Residual Excess Mortality vs. Economic Damage

Figure S.6 compares COVID mortality and the residual excess mortality, after removing COVID mortality, to economic damages; in both cases, there is a statistically significant and positive relationship with economic damage. We omit the 2 states (ND and CT) for which the CDC estimates that less than 90% of April 2020 deaths were reported by July 1, 2020.

Figure S.6: Comparisons with Economic Damage

(a) COVID Mortality vs. Economic Damage

(b) (Excess Mortality - COVID Mortality) vs. Economic Damage

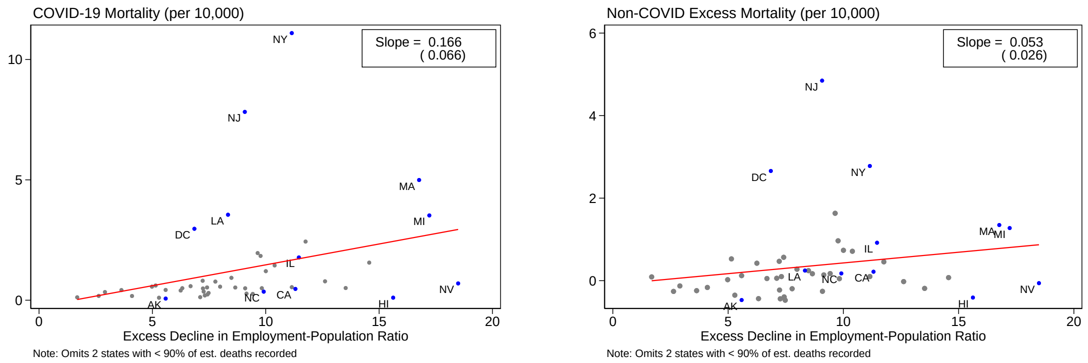

Notes: These figures plot, for each state, (a) COVID mortality and (b) residual excess mortality in April 2020 against the excess decline in the employment-population ratio in April 2020. Two states - North Dakota and Connecticut - are excluded because less than 90% of April 2020 deaths are estimated to be reported as of July 1, 2020. The red line shows the line of best fit, with the slope and standard error shown in the upper right.

7.2 Results for Non-COVID Causes of Death

The exhibits in this section show results by cause of death where COVID-19 is not listed as the underlying cause of death. Data from 2019-2020 come from the NCHS's "Weekly Counts of Deaths by State and Select Causes" file (Link: https://data.cdc.gov/NCHS/Weekly-Counts-of-Deaths-by-State-and-Select-Causes/muzy-jte6); these data are weekly and are aggregated to the month level by assuming that deaths are equally distributed across days of the week. Monthly death data from 2011-2018 come from the CDC Wonder database. We show results for the following causes of death:

- Unnatural deaths: (All-cause deaths - natural deaths) from 2019-2020 and ICD-10 codes V01-Y89 (corresponding to external causes) from 2011-2018
- Pneumonia and Influenza deaths (Non-COVID underlying cause): ICD-10 codes J10-J18
- Cancer deaths: ICD-10 codes C00-C97
- Heart disease deaths: ICD-10 codes I00-I09, I11, I13, I20-I51
- Chronic lower respiratory deaths: ICD-10 codes J40-J47
- Cerebrovascular deaths: ICD-10 codes I60-I69
- Alzheimer's deaths: ICD-10 code G30
- Diabetes deaths: ICD-10 codes E10-E14

We note the following limitations in interpreting the results from this section, which should be viewed as preliminary due to potential lags in the reporting of deaths from recent weeks:

1. Unlike our all-cause mortality estimates, the CDC does not provide a version of these death counts that accounts for historical under-reporting, so these death counts are uncorrected and represent a lower bound on deaths.

2. For 2019-2020, only weekly data are available, and these data are masked for weeks where a state had less than 10 deaths in a category. Therefore, for some causes of death, samples at the state level do not include all states (We omit any state from the sample for a cause of death if its death count from that cause is ever masked from 2019-2020).

3. These death counts are not mutually exclusive from COVID death counts because these are based on the underlying cause of death, and it is possible that COVID is not always listed as the underlying cause.

Figure S.7 shows results in April 2020 separately by cause of death, indicating that some non-COVID causes of death had positive excess mortality in April 2020 while others had negative excess mortality. Figure S.8 plots excess mortality from unnatural causes in April 2020 against the excess decline in the employment-population ratio by state, showing that there is a negative but insignificant relationship between economic damage and excess mortality from unnatural causes. Figure S.9 shows predictions and observed values in April of each year nationally for each non-COVID cause of death. For each cause of death, trends fit well prior to 2020, with the possible exception of unnatural deaths, which begins to level off around 2016; nevertheless, the observed divergence in April 2020 is visibly much larger than any prediction error.

Figures S.10 and S.11 show dotplots of excess mortality by cause of death, for each state with a sufficiently large number of deaths to have data in all months. While patterns in the sign of excess deaths tend to be consistent across states, these figures illustrate substantial heterogeneity in magnitudes. For example, New York and New Jersey, which have the largest recorded all-cause mortality and were particularly hard hit by COVID deaths, also have the largest increases in lower respiratory mortality, diabetes mortality, pneumonia and influenza mortality, and heart disease mortality.

Figure S.7: Excess Mortality and Deaths by Non-COVID Cause

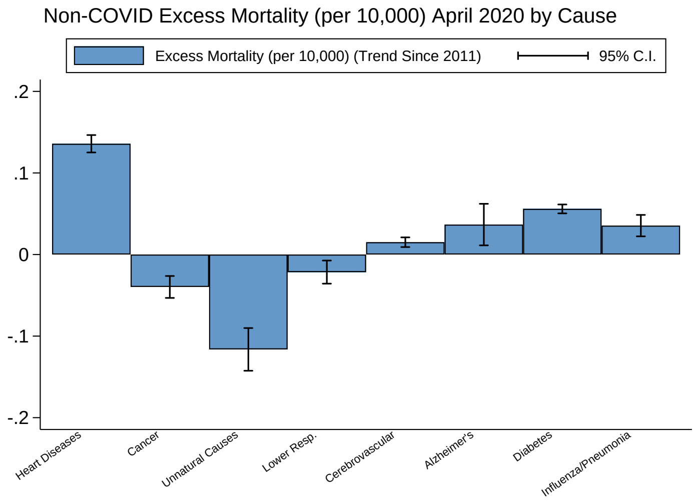

Notes: This figure shows estimated excess mortality by cause in April 2020, using a linear time trend during the pre-period 2011-2019 with month fixed effects separately for each listed cause of death. Causes of death are defined based on the ICD-10 codes listed in Section 7.2, and do not represent all non-COVID causes of death. 95% confidence intervals are shown on the estimates for each cause of death. Causes of death are ordered based on observed mortality rates in April 2020.

Figure S.8: Excess Mortality from Unnatural Causes versus Economic Damages: April 2020

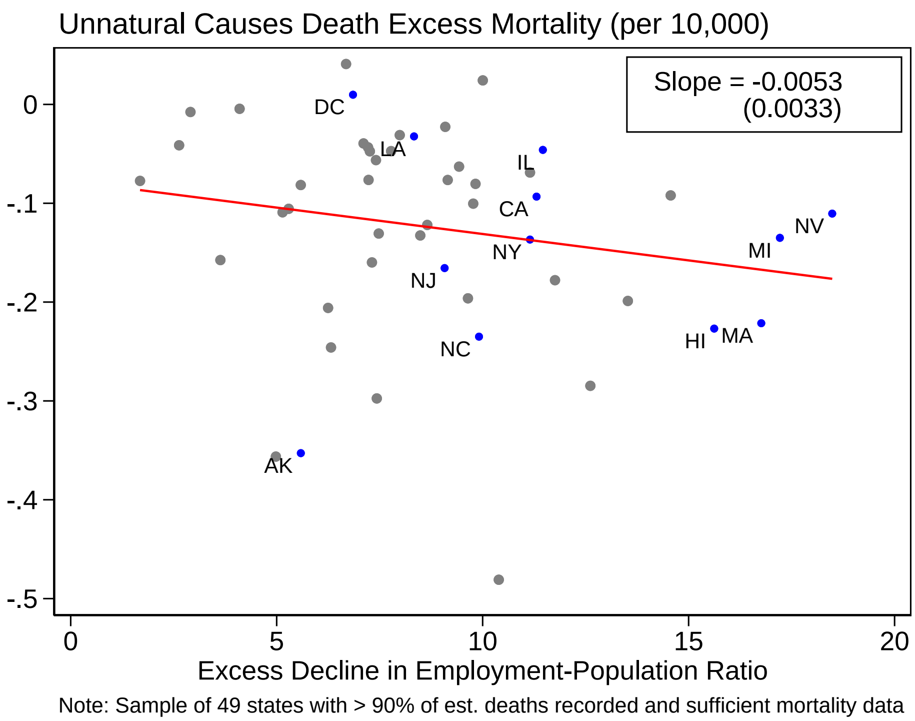

Notes: This figure plots, for each state, excess mortality from unnatural causes in April 2020 against the excess decline in the employment-population ratio in April 2020. Two states - North Dakota and Connecticut - are excluded because less than 90% of April 2020 deaths are estimated to be reported as of July 1, 2020. The red line shows the line of best fit, with the slope and standard error shown in the upper right.

Figure S.9: April Time Series Across Years: Causes of Death

(a) Unnatural Deaths

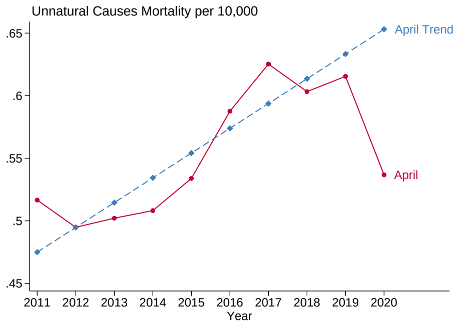

(b) Pneumonia and Influenza Deaths

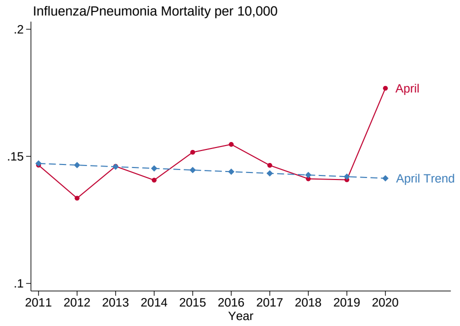

(c) Cancer Deaths

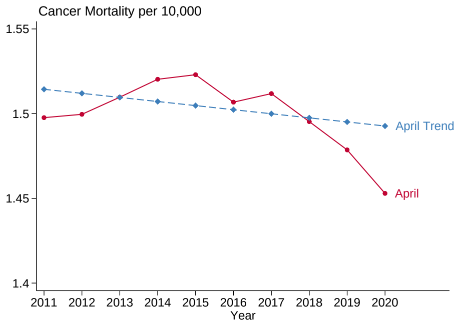

(d) Heart Disease Deaths

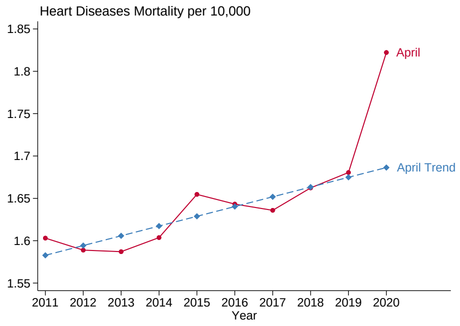

(e) Lower Respiratory Deaths

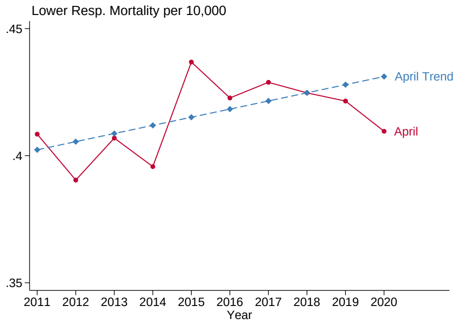

(f) Diabetes Deaths

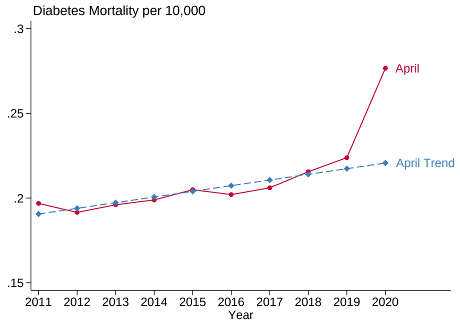

(g) Cerebrovascular Deaths

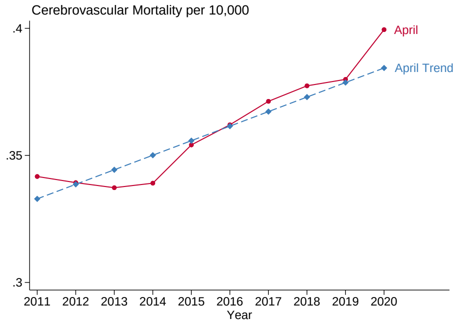

(h) Alzheimer's Deaths

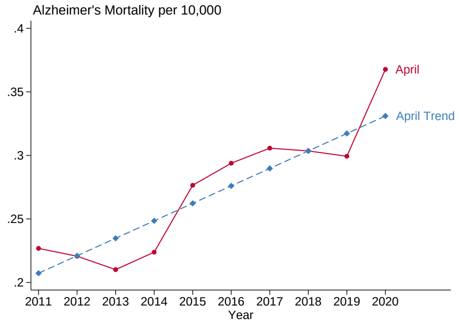

Notes: These figures show the time series for the mortality per 10,000 in April of each year, where each panel is a different cause of death. Predictions are calculated within each cause of death using a linear time trend with month fixed effects during the pre-period 2011-2019.

Figure S.10: State Heterogeneity Dotplots by Cause of Death Part 1

(a) Unnatural Deaths

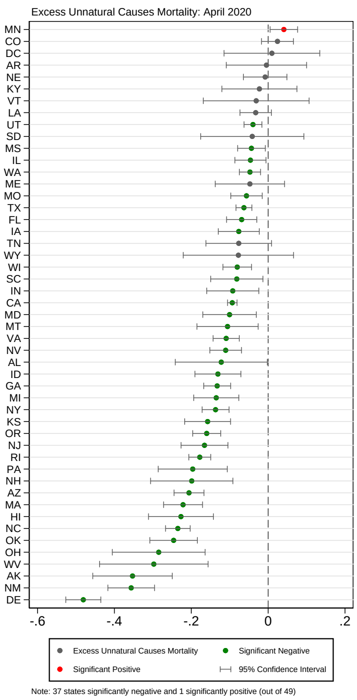

(b) Pneumonia and Influenza Deaths

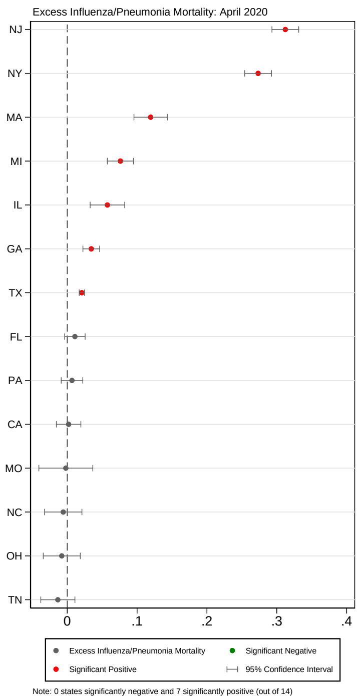

(c) Cancer Deaths

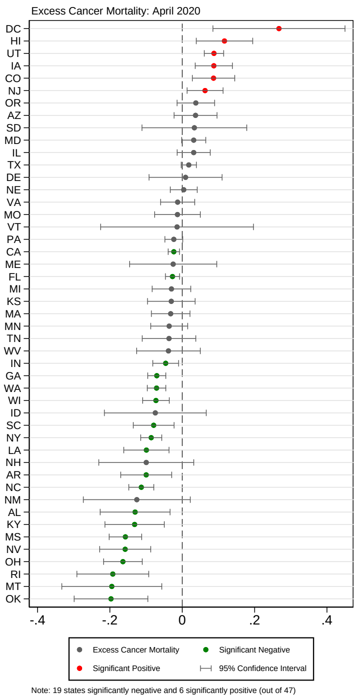

(d) Heart Disease Deaths

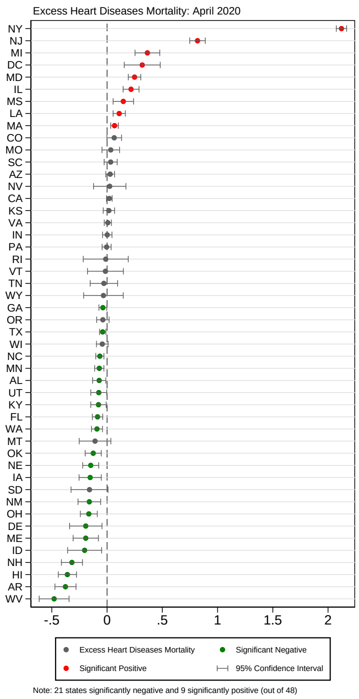

Notes: These figures show dotplots of excess mortality by cause of death and state, excluding North Dakota and Connecticut as well as states that do not have a sufficiently large number of deaths from the cause of death to have data available for each month. Heteroskedasticity-robust standard errors are used to calculate 95% confidence intervals. Point estimates are shown in red for significant positive excess mortality and in green for significant negative excess mortality.

Figure S.11: State Heterogeneity Dotplots by Cause of Death Part 2

(a) Lower Respiratory Deaths

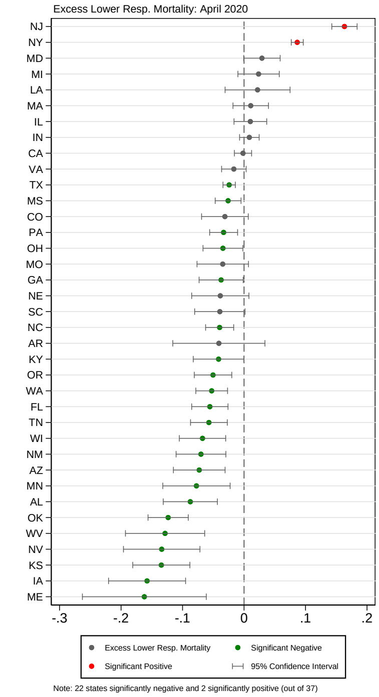

(b) Diabetes Deaths

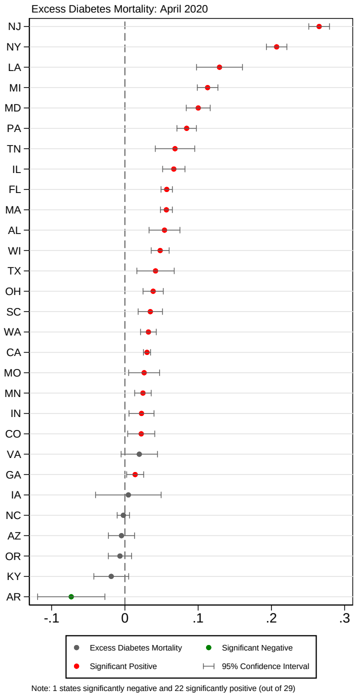

(c) Cerebrovascular Deaths

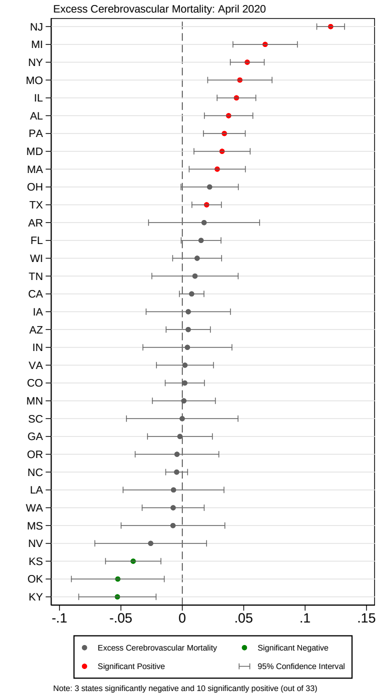

(d) Alzheimer's Deaths

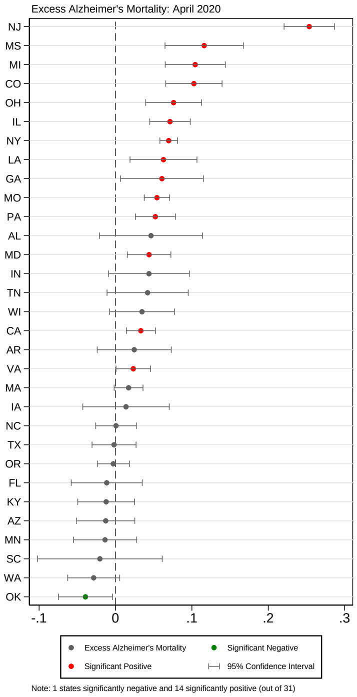

Notes: These figures show dotplots of excess mortality by cause of death and state, excluding North Dakota and Connecticut as well as states that do not have a sufficiently large number of deaths from the cause of death to have data available for each month. Heteroskedasticity-robust standard errors are used to calculate 95% confidence intervals. Point estimates are shown in red for significant positive excess mortality and in green for significant negative excess mortality.

References

[1] L. Montenovo et al., Determinants of Disparities in Covid-19 Job Losses. doi:10.3386/w27132 (May 2020).

[2] T.T.M. Bui, P. Button, E. Picciotti, Early Evidence on the Impact of COVID-19 and the Recession on Older Workers. doi:10.3386/w27448 (June 2020).

[3] D. Weinberger et al., Estimation of Excess Deaths Associated With the COVID-19 Pandemic in the United States, March to May 2020. *JAMA Internal Medicine*. doi:10.1001/jamainternmed.2020.3391 (2020).

[4] S. Woolf et al., Excess Deaths from COVID-19 and Other Causes, March-April 2020. *JAMA*. doi:10.1001/jama.2020.11787. (2020).
